# Communication development of a child with autism, the son of deaf parents, using alternative communication: a case report

**DOI:** 10.1590/2317-1782/e20250080en

**Published:** 2026-04-10

**Authors:** Cassio Kennedy de Sá Andrade, Ana Cristina de Albuquerque Montenegro, Pâmela Pontes dos Santos, Adriana Di Donato Chaves, Rafaella Asfora Siqueira Campos Lima, Isabelle Cahino Delgado, Giorvan Ânderson dos Santos Alves

**Affiliations:** 1 Programa de Pós-graduação em Linguística, Universidade Federal da Paraíba – UFPB - João Pessoa (PB), Brazil.; 2 Departamento de Fonoaudiologia, Universidade Federal de Pernambuco – UFPE - Recife (PE), Brasil.; 3 Programa Associado de Pós-graduação em Fonoaudiologia, Universidade Federal da Paraíba – UFPB - João Pessoa (PB), Brasil.; 4 Departamento de Psicologia, Inclusão e Educação, Universidade Federal de Pernambuco – UFPE - Recife (PE), Brasil.

**Keywords:** Autism Spectrum Disorder, Speech, Language and Hearing Sciences, Deafness, Child Language, Social Inclusion, Self-Help Devices

## Abstract

**Purpose:**

The main objective of this study is to describe the development of communicative skills in a 5-year-old child with autism spectrum disorder (ASD), the son of deaf parents, through an augmentative and alternative communication (AAC) system using the DHACA method (Development of Communication Skills in Autism) with an adapted AAC resource using pictograms associated with the respective signs in Libras (Brazilian Sign Language).

**Methods:**

The Autism Treatment Evaluation Checklist (ATEC) was applied for assessment. The Communication Assessment in ASD (ACOTEA) was used for direct assessment with the child.

**Results:**

The analysis of both qualitative and quantitative data from the speech-language-hearing intervention using DHACA demonstrated significant progress in the child's expressive and receptive communication, with an increase in oral productions, including verbalization of words, use of gestures, and improved shared attention. A decrease in inappropriate behaviors, such as tantrums, was also observed. This demonstrates the effectiveness of the DHACA method in implementing an AAC system in a child with ASD, the son of deaf parents.

**Conclusion:**

The study is innovative in that it proposes the functional use of the bilingual DHACA book, promoting greater interaction and use of AAC between the child and his parents.

## Introduction

Individuals with autism spectrum disorder (ASD), when unable to develop oral language, need to resort to other forms of communication that enable them to interact with their peers. In this aspect, augmentative and alternative communication (AAC) aims to promote communication accessibility between individuals with communication disorders and their interlocutors, prioritizing the information, objectives, and desires to be conveyed, using varied and equally favorable forms for the communicative act^(1)^.

AAC consists of a communication support resource that uses signals, gestures, and graphic and visual resources to develop, complement, or replace oral language when it is compromised or absent. Such systems have been developed to benefit individuals with various neurological, sensory problems and syndromes that impair speech development, such as non-progressive chronic encephalopathy, hearing impairment, ASD, and so forth^(2)^.

Authors highlight that graphic symbols and photos facilitate the understanding of information, thus improving the ability of people with ASD and speech difficulties to express their desires and needs according to the context^(3)^.

The DHACA (Development of Communication Skills in Autism) method, based on sociopragmatic theory^(4)^, aims to develop communicative skills using AAC to expand the communication of subjects with ASD, using playful activities planned according to the child's preferences^(3)^.

Case studies of children with ASD who underwent intervention using the DHACA method demonstrated the development of functional communication associated with improved behavior^(5-7)^.

A non-oral child with ASD, the son of deaf parents fluent in Libras (Brazilian Sign Language), has a significant communication and interaction gap. The study proposes an intervention with the DHACA method to develop his communication, enabling more effective interaction with his parents. It is understood that the child must learn to use communication through AAC and incorporate Libras to meet family and social needs, which can result in the development of speech^(7)^.

Thus, the main objective of this study is to describe the development of the communicative skills of a 5-year-old child with ASD, the son of deaf parents, through an AAC system with DHACA method^(5)^, an adapted AAC resource using pictograms associated with the respective signs in Libras.

The use of AAC with pictograms and Libras through the DHACA method, coupled with speech-language-hearing (SLH) therapy, is expected to promote the development of the child's communication in general, enabling interaction with his deaf parents and a greater number of interactions inside and outside the clinical setting.

## CLINICAL CASE PRESENTATION

This study was conducted by the Center for Studies in Language and Stomatognathic Functions (NELF-UFPB), in partnership with the Federal University of Pernambuco (UFPE), within the scope of the outreach program, “Autism Communicates”, and the research project, “SLH Therapy and Autism: Knowing, Intervening, and Including”. The project was approved by the Research Ethics Committee of the UFPE’s Health Sciences Center (CEP/CCS/UFPE), under number 66933317.9.0000.5208.

The study participant is a 5-year-old male child, identified as “L.”, diagnosed with a medical report of ASD, level 2 support, non-oral, without associated comorbidities. L. is in preschool at a private institution. His parents, both deaf, use Libras as their main form of communication. The father, 39, has some college education and works as a salesman; the mother, 35, has completed high school and has a technical degree in administration, working in that field.

The assessment used the Autism Treatment Evaluation Checklist (ATEC) together with the parents^(8)^. The direct assessment with the child used the Communication Assessment in ASD (ACOTEA)^(5)^. The child was assessed through playful and symbolic activities, involving action and reaction tasks, imitation, joint attention, and social interaction.

During the sessions, data were collected through records on progress record sheets (PRS) and filming; 25 videos were analyzed, five recorded in the therapy sessions, and 20 provided by the parents, totaling 3 h and 39 min. The analysis prioritized interactions that evidenced communicative skills, joint attention, and AAC use.

The PRS recorded session objectives, materials used, and patient progress. Statistical analysis of the data collected in the PRS was performed, allowing for detailed analysis of behaviors and evaluation of the impact of AAC on L's linguistic development. Initially, before using the DHACA method, the child underwent 29 sessions of an AAC approach that used a board with pictures attached with Velcro, where the child had to hand over pictures to request something. This was later replaced by the DHACA method, using the DHACA communication book, which integrates pointing to pictograms as a strategy to convey messages.

## Discussion

This study highlights the contributions of the DHACA method and the use of the communication book in the development of L.'s communicative skills, demonstrating the importance of AAC in the therapeutic context of ASD.

The results are presented according to the assessment protocols (ATEC^(8)^ and ACOTEA^(5)^) used before and after the intervention, the analysis of 29 records extracted from the PRS, behavioral data from SLH therapy sessions, and qualitative observations made during the 210-day clinical follow-up, divided into 30-day intervals.

In addition to the DHACA essential vocabulary page with individual pictograms to develop the method's first skill, the approach for this research used the traditional DHACA AAC book and the bilingual DHACA book (pictograms associated with Libras). The progressive choice of these resources aimed to meet the child's communicative needs, integrating parents into the process through Libras.

Three types of physical AAC resources were implemented during the intervention:

1. DHACA essential vocabulary page and individual pictograms: used in the first 7 sessions (24% of the total).
2. Traditional DHACA book: applied in 13 sessions (45%), allowing for greater functional interaction.
3. Bilingual DHACA book: used in the last nine sessions (31%), promoting family inclusion and language transition.

**Figure 1:**
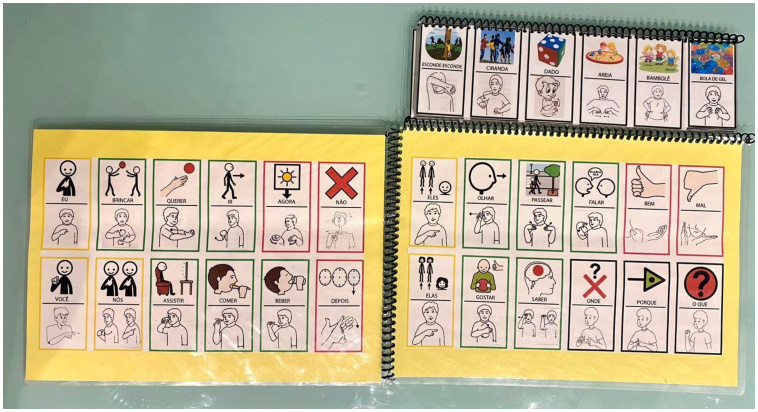
Bilingual DHACA book

The use of the traditional DHACA communication book was the main AAC resource used for AAC implementation. The DHACA method was used in two phases, initially using the traditional DHACA communication book and then switching to the bilingual version of the DHACA book with pictures described through traditional pictograms along with Libras signs (Figure 1).

The decision to adapt the traditional DHACA book to a bilingual version emerged from the possibility of increasing family participation by introducing AAC, using Libras as L.'s mother tongue and Portuguese as a second language. This approach broadened communicative possibilities, allowing the child to interact in two linguistic fields: the signed (Libras) and the oral (Portuguese).

Three main skills were worked on during the interventions^(9)^:

1. Initial communicative intention: addressed in 6.9% of sessions.
2. Request with lexical expansion in accessory vocabulary: worked on in 10.3% of sessions.
3. Request with lexical and morphosyntactic expansion: focus in 82.8% of sessions.

The third skill mentioned above was central to L.'s communicative development, allowing the formation of phrases such as “I want a ball” by pointing to pictures in the bilingual communication book.

It is worth mentioning that whenever the child pointed to the book to form sentences, the therapist reinforced it by saying aloud what the picture was indicating to develop the child's oral skills.

This skill was developed with greater emphasis when traditional and bilingual DHACA books were used. The transition to the bilingual book also reinforced vocabulary expansion and joint attention, as observed in the PRS, thanks to the inclusion of parents and the use of Libras^(10)^.

The PRS further recorded that, in addition to verbal stimuli provided by the therapist during SLH therapy sessions, cues were also used in almost all sessions, assisting the child in handling the AAC book. Cues were recorded in 93.1% of the sessions, presenting variations such as physical, verbal, and visual cues and all their possible combinations. All these cues help the child develop the skills necessary to use the AAC book and are generally used to initiate interactions with the child. Physical cues are those that mainly involve touch, visual cues involve the demonstration of figures in the AAC book, and verbal cues are the speech of the communication partner^(11)^.

The results obtained from L. revealed important challenges and progress:

- Tantrums: occurred in 51.7% of sessions, mainly in their initial moments, related to separation from parents.
- Eye contact: present in 79.3% of sessions, being essential for joint attention and social interactions.
- Vocalizations and verbalizations:
  - Vocalizations (incomprehensible sounds): 61.2% of sessions.
  - Verbalizations (comprehensible words): 34.5%, but still predominantly monosyllabic.
  - Spontaneous speech: 13.8%, limited, but with progress during the use of the bilingual book.

Temporal analysis, corresponding to the set of observations collected over a specific time interval, was performed to complement these observations. This analysis was used to observe the child's development during SLH therapy, mainly considering the resources, communicative skills, type of prompt, eye contact, vocalizations, verbalizations, echolalia, spontaneous speech, stimulator, and skills acquired throughout the 29 sessions, which were divided into 30-day sets, totaling the 210 days the child spent under observation at the SLH clinic. It is important to mention that the intervals corresponding to the end-of-year break are not included in the time series.

1. **Resources and development of communication skills**: The first skill of the DHACA method was initially stimulated with the essential vocabulary page and individual pictograms. Then, the focus shifted to sentence construction with the traditional DHACA book, corresponding to the skill, “Request with lexical and morphosyntactic expansion”. Next, using the bilingual DHACA book, the development of the third skill was stimulated with vocabulary expansion and social interaction, evidenced by increased verbalization and eye contact.
2. **Cues and encouragement:** Physical, visual, and verbal cues were used in 93.1% of the sessions, often in combination, which was essential to facilitate interaction and learning how to use the communication book.

The results show that the DHACA book effectively increased the child's communicative skills, especially when compared to the previously used AAC resource with loose pictures. The adoption of the bilingual DHACA book also contributed considerably to this qualitative leap, in which the child begins to manifest spontaneous speech. This data mainly points to the contribution of sign language in this process, which enabled the inclusion of parents in the AAC implementation process. With increased interaction, it is natural for the child to develop their linguistic skills and begin to present more effective communicative ways to interact with their interlocutors^(12)^.

The traditional DHACA book was expected to improve the child's communication, but the great leap occurred by using the DHACA communication book with Libras, in which the child could receive verbal stimuli from their hearing partners and signed stimuli from their deaf parents. This richness of stimuli led to a very significant communicative evolution in a short time. As previously mentioned, only nine therapeutic sessions used the bilingual book, which was sufficient to improve the child's communicative performance as a whole^(12)^.

With the increase in the child's repertoire and the use of AAC, a modification is noted in “non-functional” aspects such as tantrums, as well as an increase in social communication and interaction. Given these findings, it is possible to infer how much the child could have evolved communicatively if the bilingual book had been adopted from the first moment of the intervention. However, considering the participant's age and the observed path, it is understood that the first sessions, as conducted, may have played an important role in approaching the method, contributing to the preparation of the child and their family for the introduction of Libras, included in the AAC book, reinforcing an inclusive and joint action between professional and family^(6)^.

Seven sessions were held after the start of SLH therapy, followed by the end-of-year break at the clinic-school and the consequent pause in services. Nevertheless, parents were strongly advised to continue the activities, mainly using the communication book that had been adopted and was being used during the sessions with the SLH pathologist.

The resumption of activities the following year was marked by a new assessment of the child, this time using ATEC, whose objective is to evaluate the effectiveness of the treatment adopted for the patient. The child's ATEC score was 89 points, and after 9 months of treatment with 20 SLH sessions, he was reassessed with ATEC at the end of the year, obtaining a score of 59. The lower the score, the fewer the problems; hence, this 30-point drop from the previous assessment indicates the effectiveness of the intervention (Table 1).

**Table 1:** ATEC results

Subscale	1st Assessment (February 21, 2019)	2nd Assessment (December 5, 2019)	Difference
Speech/Language/Communication	23	21	−2
Sociability	24	16	−8
Sensory/Cognitive Skills	15	14	−1
Health/Physical Aspects/Behavior	27	8	−19
Total Sum	89	59	-30

The scores in the two ATEC assessment results point to a significant improvement in Subscale IV, which corresponds to health, physical, and behavioral aspects. It is also important to highlight the significant improvement obtained in​​sociability, which corresponds to Subscale II.

As described earlier, the child was assessed with ACOTEA and reassessed after 8 months of treatment. During the assessment, L. presented typical characteristics of ASD, such as difficulties in social interaction, restricted vocabulary, reduced eye contact, and little communicative initiative, although he could understand and execute simple commands (Table 2).

**Table 2:** ACOTEA results

Category	1st Assessment (March 7, 2019)	2nd Assessment (November 21, 2019)	Difference
Expressive Communication	9	11.5	+2.5
Receptive Communication	0	3	+3
Social Behavior	2	5.5	+3.5
Total Sum	11	20	+9

The ACOTEA scores indicate an evolution in all categories, which is consistent with what has already been mentioned regarding ATEC. Based on these results, it can be stated that the SLH intervention using the DHACA method was effective and improved the patient's communicative skills. It is important to mention that, in addition to the appropriate intervention, the participation of the family as facilitators and encouragers of interaction through the bilingual DHACA book contributed significantly to achieving this result.

It is already known that individuals with ASD have impaired communication and forms of socialization, so much so that, when they are referred to SLH therapy, they are offered the possibility of improving their communicative abilities. The use of AAC in sessions with a SLH pathologist aims to establish and/or improve the functional communication of individuals with communication problems; in the case of ASD, non-oral systems work quite satisfactorily to establish functional communication^(6)^.

The introduction of the bilingual DHACA book was a milestone in L.'s progress, including his parents, and improving social and functional communication. The evolution of L.'s communicative skills demonstrates the effectiveness of the DHACA method and bilingual AAC, highlighting the importance of interventions that integrate multiple communication partners.

The study has some limitations, as it was restricted to a single subject, which prevents the generalization of the results. Therefore, future studies with a larger sample and in different contexts are necessary.

## FINAL COMMENTS

The study points to the effectiveness of the DHACA method in implementing an AAC system in a child with ASD, the son of deaf parents. The study is innovative in that it proposes the functional use of the bilingual DHACA book, promoting greater interaction and use of AAC between the child and his parents.

The analysis of both qualitative and quantitative data from the SLH intervention using DHACA demonstrated significant progress in the child's expressive and receptive communication, with an increase in oral productions, including the verbalization of words, the use of gestures, and improved shared attention. A decrease in inappropriate behaviors, such as tantrums, was also observed.

The contributions of this study are relevant, especially to SLH therapy, by demonstrating that the DHACA method can be an effective tool for the development of communicative skills in children with ASD. Continued research in this area, focusing on multimodal interactions and AAC use, could broaden our understanding of best practices for intervention in these cases. Thus, this work opens possibilities for new investigations that can explore the potential of DHACA in other contexts and with different populations, contributing to a more comprehensive understanding of language development in children with communication difficulties.
