# Supplementary material for: Communication development of a child with autism, the son of deaf parents, using alternative communication: a case report
Source: Codas. 2026 Apr 10;38(2):e20250080. doi: 10.1590/2317-1782/e20250080en (PMC13075831; doi:10.1590/2317-1782/e20250080en)
Supplement: Translation

# Translation: Desenvolvimento da comunicação de uma criança autista, filha de pais surdos com uso de comunicação alternativa: relato de caso

**DOI:** 10.1590/2317-1782/e20250080pt

**Authors:** Cassio Kennedy de Sá Andrade, Ana Cristina de Albuquerque Montenegro, Pâmela Pontes dos Santos, Adriana Di Donato Chaves, Rafaella Asfora Siqueira Campos Lima, Isabelle Cahino Delgado, Giorvan Ânderson dos Santos Alves

**Affiliations:** 1 Programa de Pós-graduação em Linguística, Universidade Federal da Paraíba – UFPB - João Pessoa (PB), Brazil.; 2 Departamento de Fonoaudiologia, Universidade Federal de Pernambuco – UFPE - Recife (PE), Brasil.; 3 Programa Associado de Pós-graduação em Fonoaudiologia, Universidade Federal da Paraíba – UFPB - João Pessoa (PB), Brasil.; 4 Departamento de Psicologia, Inclusão e Educação, Universidade Federal de Pernambuco – UFPE - Recife (PE), Brasil.

**Keywords:** Transtorno do Espectro Autista, Fonoaudiologia, Surdez, Linguagem Infantil, Inclusão Social, Tecnologia Assistiva

## Abstract

**Objetivo:**

O objetivo principal deste estudo é descrever o desenvolvimento das habilidades comunicativas de uma criança com Transtorno do Espectro Autista (TEA), com idade de 5 anos, filha de pais surdos, por meio do uso de um sistema de comunicação aumentativa e alternativa utilizando o método DHACA - Desenvolvimento das Habilidades de Comunicação no Autismo com um recurso de Comunicação Alternativa e Aumentativa (CAA) adaptado com uso de pictogramas associados aos respectivos sinais em LIBRAS.

**Método:**

Para a avaliação, foi aplicado o protocolo ATEC (Autism Treatment Evaluation Checklist). E, na avaliação direta junto a criança, o ACOTEA (Avaliação da Comunicação no Transtorno do Espectro do Autismo).

**Resultados:**

A análise dos dados, tanto qualitativos quanto quantitativos, da intervenção fonoaudiológica com uso do DHACA, demonstrou avanços significativos na comunicação expressiva e receptiva da criança, com aumento de produções orais incluindo a verbalização de palavras, o uso de gestos, além da melhora da atenção compartilhada. Também, foi observado diminuição de comportamentos inadequados, como as birras. Sendo assim, foi possível apresentar a efetividade do método DHACA na implementação de um sistema de CAA em uma criança com TEA, filha de pais surdos.

**Conclusão:**

O estudo inova, quando propõe a utilização funcional do livro DHACA bilíngue, promovendo maior interação e uso de CAA entre criança e seus pais.

## INTRODUÇÃO

Os sujeitos com Transtorno do Espectro Autista (TEA), quando não conseguem desenvolver a linguagem oral, precisam recorrer a outras formas de comunicação que lhes ofereça a possibilidade de interagir com seus pares. E neste aspecto, a Comunicação Aumentativa e Alternativa (CAA) tem como objetivo principal promover acessibilidade comunicacional entre a pessoa com transtornos na comunicação e o seu interlocutor, de modo que seja priorizada a informação, os objetivos e o desejo que se pretende transmitir utilizando, para tanto, formas variadas e igualmente favoráveis ao ato comunicativo^(1)^.

A CAA consiste em um recurso de apoio à comunicação no qual as sinalizações, gestos, recursos gráficos e visuais são utilizados como forma de desenvolver, complementar ou substituir a linguagem oral, quando estiver comprometida ou ausente. Tais sistemas vêm sendo desenvolvidos para beneficiar indivíduos com diferentes problemas neurológicos, sensoriais e síndromes que prejudicam o desenvolvimento da fala, tais como Encefalopatia Crônica não progressiva, Deficiência Auditiva, TEA e outros^(2)^.

Autores destacam que a utilização de símbolos gráficos ou fotos facilitam a compreensão de informações e, por seguinte, melhoram a capacidade da pessoa com TEA e com dificuldades na fala, de expressar seus desejos e necessidades de acordo com o contexto^(3)^.

O uso do método DHACA, baseado na teoria sociopragmática^(4)^ objetiva o desenvolvimento de habilidades comunicativas com o uso da CAA, de modo a ampliar a comunicação de sujeitos com TEA, com uso de atividades lúdicas planejadas com base nas preferências da criança^(3)^.

Estudos de caso de crianças com TEA que foram submetidas a intervenção com uso do método DHACA, demonstraram desenvolvimento de uma comunicação funcional associada com melhora no comportamento^(5-7)^.

Diante de um caso de uma criança com TEA não verbal, filha de pais surdos, fluentes em LIBRAS, há uma lacuna significativa no processo de comunicação e interação. O estudo propõe uma intervenção com método DHACA com objetivo de desenvolver a comunicação, possibilitando a interação com os pais de forma mais efetiva. Entende-se que para atender a demanda familiar e social, a criança deve aprender a utilizar a comunicação através da CAA e inserir a Libras neste processo, que pode resultar no desenvolvimento da fala^(7)^.

Sendo assim, o objetivo principal deste estudo é descrever o desenvolvimento das habilidades comunicativas de uma criança com TEA, com idade de 5 anos, filha de pais surdos, por meio do uso de um sistema de comunicação aumentativa e alternativa utilizando n método DHACA - Desenvolvimento das Habilidades de Comunicação no Autismo^(5)^ com um recurso de CAA adaptado com uso de pictogramas associados aos respectivos sinais em LIBRAS.

É esperado que com o emprego da CAA com pictogramas e sinais de LIBRAS por meio do método DHACA atrelado à terapia fonoaudiológica promova o desenvolvimento da comunicação da criança de modo geral, possibilitando a interação com os pais surdos, bem como maior número de interações, que podem ocorrer tanto no contexto da clínica como fora dela.

## APRESENTAÇÃO DO CASO CLÍNICO

Este estudo foi realizado pelo Núcleo de Estudos em Linguagem e Funções Estomatognáticas (NELF-UFPB), em parceria com a Universidade Federal de Pernambuco (UFPE), no âmbito do projeto de extensão “Autismo Comunica” e do projeto de pesquisa “Fonoaudiologia e Autismo: conhecer, intervir e incluir”. O projeto foi aprovado pelo Comitê de Ética em Pesquisa do Centro de Ciências da Saúde da UFPE (CEP/CCS/UFPE), sob nº 66933317.9.0000.5208.

O participante do estudo é uma criança do sexo masculino, identificada como “L.”, de 5 anos, diagnosticada com laudo médico: Transtorno do Espectro do Autismo (TEA), nível 2 de suporte, não-verbal. sem a presença de comorbidades associadas. L. está na primeira fase do Ensino Infantil em uma escola privada. Seus pais, ambos surdos, utilizam Libras como principal forma de comunicação. O pai, de 39 anos, possui ensino superior incompleto e trabalha como vendedor; a mãe, de 35 anos, tem ensino médio completo e curso técnico administrativo, exercendo a profissão na área.

Para a avaliação, foi aplicado, juntos aos pais, o protocolo ATEC (*Autism Treatment Evaluation Checklist*)^(8)^. E, na avaliação direta junto a criança, o ACOTEA (Avaliação da Comunicação no Transtorno do Espectro do Autismo)^(5)^. As avaliações junto à criança foram realizadas por meio de atividades lúdicas e simbólicas, envolvendo tarefas de ação e reação, imitação, atenção conjunta e interação social.

Além disso, durante as sessões, os dados eram coletados por meio de registros nas fichas de registro de evolução (FRE) e filmagens. Foram analisados 25 vídeos, sendo 5 gravados nas sessões terapêuticas e 20 fornecidos pelos pais, totalizando 3 horas e 39 minutos. A análise priorizou interações que evidenciassem habilidades comunicativas, atenção conjunta e uso da Comunicação Aumentativa e Alternativa (CAA).

As fichas de registro de evolução (FRE) registraram objetivos das sessões, materiais utilizados e evolução do paciente. Foi realizada análise estatística dos dados coletados na FRE que permitiu detalhar comportamentos e avaliar o impacto da CAA no desenvolvimento linguístico de L. Inicialmente, antes do uso do método DHACA, a criança foi submetida a 29 sessões uma proposta de CAA que utilizava uma prancha com figuras presas com velcro em que a criança deveria realizar a entrega de figuras para solicitar algo. Posteriormente foi substituída pelo método DHACA, utilizando assim o livro de comunicação DHACA, que integra o apontar os pictogramas como estratégia para transmitir as mensagens.

## DISCUSSÃO

Este estudo destaca as contribuições do método DHACA e do uso do livro de comunicação no desenvolvimento das habilidades comunicativas de L., evidenciando a importância da CAA no contexto terapêutico do TEA.

Os resultados apresentados baseiam-se nos protocolos avaliativos (ATEC^(8)^ e ACOTEA^(5)^) utilizados antes e após intervenção, na análise de 29 registros extraídos das fichas de registro de evolução (FRE), dados comportamentais das sessões fonoaudiológicas e observações qualitativas feitas durante o período de 210 dias de acompanhamento clínico, dividido em intervalos de 30 dias.

A abordagem para esta pesquisa utilizou como recursos de CAA, além da página de vocabulário essencial DHACA com pictogramas avulsos no desenvolvimento da primeira habilidade do método, o livro DHACA tradicional de CAA, e, por fim, o uso do livro DHACA bilíngue (pictogramas associados a Libras). A escolha progressiva desses recursos visou atender às necessidades comunicativas da criança, integrando os pais no processo por meio da Libras.

Foram implementados três tipos de recursos físicos de CAA durante a intervenção

1. Página de vocabulário essencial DHACA e pictogramas avulsos: utilizada nas 7 primeiras sessões (24% do total).
2. Livro DHACA tradicional: aplicado em 13 sessões (45%), permitindo maior interação funcional.
3. Livro DHACA bilíngue: utilizado nas 9 últimas sessões (31%), promovendo inclusão familiar e transição linguística.

O uso do livro de comunicação DHACA tradicional foi o principal recurso de CAA utilizado para implementação da CAA. O método DHACA foi utilizado em dois momentos utilizando inicialmente o livro de comunicação DHACA tradicional e depois passando o livro DHACA com a versão bilíngue com figuras descritas com pictogramas tradicionais junto com sinais de Libras. (Figura 1)

**Figura 1:** Livro DHACA bilíngue

A decisão de adaptação do livro DHACA tradicional para bilíngue emergiu diante da possibilidade de aumentar a participação da família, apresentando a CAA, usando Libras como língua materna de L. e português como segunda língua. Essa abordagem ampliou as possibilidades comunicativas, permitindo que a criança interagisse em dois campos linguísticos: o sinalizado (Libras) e o oralizado (português).

Três habilidades principais foram trabalhadas durante as intervenções^(9)^:

1. Intenção Comunicativa Inicial: abordada em 6,9% das sessões.
2. Pedido com ampliação lexical no vocabulário acessório: trabalhada em 10,3% das sessões.
3. Pedido com ampliação lexical e morfossintática: foco em 82,8% das sessões.

A terceira habilidade mencionada acima foi central para o desenvolvimento comunicativo de L., permitindo a formação de frases como “Eu quero bola” ao apontar para figuras no livro de comunicação bilíngue.

Vale mencionar que sempre que a criança apontava para o livro para formar frases, a terapeuta reforçava o apontar dizendo em voz alta o que a figura estava indicando, com a finalidade de desenvolver a oralidade na criança.

Essa habilidade foi desenvolvida com maior ênfase nos momentos em que o livro DHACA tradicional e o bilíngue foram utilizados. A transição para o livro bilíngue também reforçou a ampliação vocabular e a atenção conjunta, conforme observado nas FRE, graças à inclusão dos pais e ao uso de Libras^(10)^.

Também foi constatado nos registros da FRE, que atreladas aos estímulos de verbalização, feitos pela terapeuta durante o atendimento fonoaudiológico, as dicas também foram utilizadas em quase todas as seções e servem como forma de auxiliar a criança no manejo do livro de CAA. As dicas foram registradas em 93,1% das sessões apresentando variações tais como: físicas, verbais e visuais e todas suas possíveis combinações. Todas essas dicas são utilizadas para ajudar a criança a desenvolver as habilidades necessárias para utilização do livro de CAA, geralmente são utilizadas para iniciar as interações com a criança. As dicas físicas são as que envolvem principalmente o toque e o tato; as dicas visuais envolvem a demonstração de figuras no livro DHACA; por fim as dicas verbais são a fala do parceiro de comunicação^(11)^.

Os resultados obtidos de L. revelaram desafios e progressos importantes:

- Birras: ocorreram em 51,7% das sessões, principalmente nos momentos iniciais das sessões, relacionados à separação dos pais
- Contato Visual: presente em 79,3% das sessões, sendo essencial para atenção conjunta e interações sociais.
- Vocalizações e verbalizações:
  - Vocalizações (sons incompreensíveis): 61,2% das sessões.
  - Verbalizações (palavras compreensíveis): 34,5%, mas ainda predominantemente monossilábicas.
  - Fala Espontânea: 13,8%, limitada, mas com progressos durante o uso do livro bilíngue.

Para complementar essas observações, foram feitas análises temporais que correspondem ao conjunto de observações coletadas ao longo de um determinado intervalo de tempo. Esta análise foi utilizada para observar o desenvolvimento da criança durante o atendimento fonoaudiológico, considerando principalmente as variáveis: recursos, habilidades comunicativas, tipo de dica, contato visual, vocalizações, verbalizações, ecolalia, fala espontânea, estimulador e habilidade adquirida ao longo das sessões 29 sessões, as quais foram divididas em conjuntos de 30 dias, totalizando os 210 dias com os quais a criança passou sobre acompanhamento na clínica de fonoaudiologia. É importante mencionar que não estão inclusos na série temporal os intervalos que correspondem ao recesso de fim de ano ocorrido no final do ano.

1. **Recursos e desenvolvimento das habilidades comunicativas**: Inicialmente, com a página do vocabulário essencial e os pictogramas avulsos, foi estimulada a primeira habilidade do método DHACA. Em seguida com o livro DHACA tradicional, focou-se na construção de frases o que corresponde a habilidade “Pedido com ampliação lexical e morfossintática”. Em seguida, com o uso do livro DHACA bilíngue foi estimulado o desenvolvimento da terceira habilidade com ampliação vocabular e interação social, evidenciadas no aumento das verbalizações e do contato visual.
2. **Dicas e Estímulos:** Foram utilizadas dicas físicas, visuais e verbais em 93,1% das sessões, muitas vezes combinadas. A combinação de dicas foi essencial para facilitar a interação e a aprendizagem do manejo do livro de comunicação.

Os resultados mostram que é possível apontar que o uso do livro DHACA foi eficaz em aumentar as habilidades comunicativas da criança, principalmente quando comparado ao recurso de CAA com figuras soltas usado anteriormente. A adoção do livro DHACA bilíngue também contribuiu consideravelmente para esse salto qualitativo, no qual a criança começa a manifestar a fala espontânea. Esse dado aponta principalmente para a contribuição da língua de sinais nesse processo, que possibilitou a inclusão dos pais no processo de implementação da CAA. Com o aumento das interações, é natural que a criança desenvolva suas habilidades linguísticas e comece a apresentar formas comunicativas mais eficazes para interagir com seus interlocutores^(12)^.

Já era esperado que o uso do livro DHACA tradicional melhorasse a comunicação da criança, mas o grande salto ocorreu quando se passa a utilizar o livro de comunicação DHACA com os sinais de LIBRAS, na qual a criança pode receber estímulos verbais de seus parceiros ouvintes, e também os estímulos sinalizados de seus pais surdos. Essa riqueza de estímulos acabou causando uma evolução comunicativa bastante significativa em pouco tempo, como se sabe, foram apenas nove sessões terapêuticas utilizando o livro bilíngue, porém, como já visto, esse tempo foi suficiente para melhorar o desempenho comunicativo da criança como um todo^(12)^.

Com o aumento de repertório infantil e o uso do CAA, nota-se uma modificação em aspectos “não funcionais” como birra e, bem como o aumento da comunicação social e da interação. Diante desses achados, é possível inferir o quanto a criança poderia ter evoluído comunicativamente se o livro bilíngue tivesse sido adotado desde o primeiro momento da intervenção. Contudo, considerando-se a idade do participante e o percurso observado, entende-se que as primeiras sessões, conforme realizadas, podem ter desempenhado um papel importante de aproximação ao método, contribuindo para a preparação da criança e de seus familiares para a introdução da Libras que foi incluída no livro de CAA, reforçando uma atuação inclusiva e conjunta entre profissional e família^(6)^.

Após o início da terapia fonoaudiológica, seguiram-se um total de sete sessões, sobrevindo então o recesso de fim de ano na clínica-escola e a consequente pausa referente aos atendimentos. Não obstante, recomendou-se firmemente aos pais dar continuidade às atividades, principalmente utilizando o livro de comunicação que fora adotado e estava sendo utilizado durante as sessões com a fonoaudióloga.

A retomada das atividades no ano seguinte foi marcada pela realização de uma nova avaliação da criança, desta feita utilizando-se o protocolo ATEC, cujo objetivo é o de avaliar a efetividade do tratamento adotado para o paciente. O *score* obtido pela criança no ATEC, fora de 89 pontos e, após 9 meses de atendimento corroborado pela realização de 20 sessões de atendimento fonoaudiológico, uma nova avaliação utilizando o ATEC fora realizada ao final do ano, obtendo o *score* 59, denotando uma queda de 30 pontos em comparação à última avaliação realizada, que significa quanto menor a pontuação, menores os problemas, portanto, esse score é indicativo de efetividade da intervenção (Tabela 1).

**Tabela 1:** Resultados do ATEC

Subescala	1ª Avaliação (21/02/2019)	2ª Avaliação (05/12/2019)	Diferença
Fala/Linguagem/Comunicação	23	21	-2
Sociabilidade	24	16	-8
Capacidade Sensorial/Cognitiva	15	14	-1
Saúde/Aspectos Físicos/Comportamento	27	8	-19
Soma Total	**89**	**59**	**-30**

Conforme os *scores* visualizados no resultado das duas avaliações com ATEC, nota-se uma melhora significativa na Subescala IV, que corresponde à saúde, aspectos físicos e de comportamento, cumprindo destacar, ainda, o aperfeiçoamento importante obtido na questão da sociabilidade, que corresponde à Subescala II.

Conforme descrito anteriormente, foi aplicado junto as crianças o protocolo ACOTEA e a reavaliação aconteceu após oito meses de tratamento. Durante a avaliação, L. apresentou características típicas do TEA, como dificuldades de interação social, vocabulário restrito, contato visual reduzido e pouca iniciativa comunicativa. No entanto, demonstrou capacidade de compreender e executar comandos simples (Tabela 2).

**Tabela 2:** Resultados do ACOTEA

Categoria	1ª Avaliação (07/03/2019)	2ª Avaliação (21/11/2019)	Diferença
Comunicação Expressiva	9	11,5	+2,5
Comunicação Receptiva	0	3	+3
Comportamento Social	2	5,5	+3,5
Soma Total	11	20	+9

De acordo com os *scores* obtidos mediante aplicação do protocolo ACOTEA, nota-se uma evolução em todas as categorias, o que coaduna com o já apresentado anteriormente, diante da aplicação do ATEC. Através desses resultados, pode-se afirmar que a intervenção terapêutica-fonoaudiológica por meio do método DHACA foi realizada de modo eficaz, a par da melhoria impressa às habilidades comunicativas do paciente. É importante mencionar que além da intervenção adequada, a participação da família, atuando como facilitadores e incentivadores da interação por meio do uso do livro DHACA bilíngue, contribuiu sensivelmente para a obtenção deste resultado.

Já é sabido que os sujeitos com TEA apresentam alterações na comunicação e nas formas de socialização, tanto que, quando esses sujeitos são encaminhados para a terapia fonoaudiológica, lhes é oferecida a possibilidade de melhorar suas capacidades comunicativas. A utilização da CAA nas sessões com fonoaudiólogo tem como objetivo estabelecer e/ou melhorar a comunicação funcional de sujeitos com problemas de comunicação, no caso do TEA a utilização de sistemas não orais funciona de maneira bastante satisfatória, de modo a possibilitar o estabelecimento de uma comunicação funcional^(6)^.

A introdução do livro DHACA bilíngue foi um marco no progresso de L., promovendo a inclusão dos pais e melhorando a comunicação social e funcional. A evolução das habilidades comunicativas de L. evidencia a eficácia do método DHACA e da CAA bilíngue, destacando a importância de intervenções que integram múltiplos parceiros de comunicação.

O estudo apresenta algumas limitações, pois foi restrito a um único sujeito, o que impede a generalização dos resultados. Portanto, futuros estudos com uma amostra maior e em diferentes contextos são necessários.

## COMENTÁRIOS FINAIS

O estudo aponta para a efetividade do método DHACA na implementação de um sistema de Comunicação Alternativa e Aumentativa (CAA) em uma criança com Transtorno do Espectro Autista (TEA), filha de pais surdos. O estudo inova, quando propõe a utilização funcional do livro DHACA bilíngue, promovendo maior interação e uso de CAA entre criança e seus pais.

A análise dos dados, tanto qualitativos quanto quantitativos, da intervenção fonoaudiológica com uso do DHACA, demonstrou avanços significativos na comunicação expressiva e receptiva da criança, com aumento de produções orais incluindo a verbalização de palavras, o uso de gestos, além da melhora da atenção compartilhada. Foi observado também diminuição de comportamentos inadequados, como as birras.

As contribuições deste estudo são relevantes, especialmente no campo da Fonoaudiologia, ao demonstrar que o método DHACA pode ser uma ferramenta eficaz para o desenvolvimento de habilidades comunicativas em crianças com TEA. A continuidade de pesquisas nessa área, com enfoque nas interações multimodais e no uso da comunicação alternativa, poderá ampliar o conhecimento sobre as melhores práticas para a intervenção nesses casos. Assim, este trabalho abre possibilidades para novas investigações, que podem explorar o potencial do DHACA em outros contextos e com diferentes populações, contribuindo para uma compreensão mais abrangente do desenvolvimento da linguagem em crianças com dificuldades de comunicação.
